# Kaposi’s Sarcoma-Associated Herpesvirus Increases PD-L1 and Proinflammatory Cytokine Expression in Human Monocytes

**DOI:** 10.1128/mBio.00917-17

**Published:** 2017-10-10

**Authors:** Kurtis M. Host, Sarah R. Jacobs, John A. West, Zhigang Zhang, Lindsey M. Costantini, Charles M. Stopford, Dirk P. Dittmer, Blossom Damania

**Affiliations:** a Lineberger Comprehensive Cancer Center, University of North Carolina at Chapel Hill, Chapel Hill, North Carolina, USA; b Department of Microbiology and Immunology, University of North Carolina at Chapel Hill, Chapel Hill, North Carolina, USA; University of Michigan—Ann Arbor

**Keywords:** Kaposi’s sarcoma-associated herpesvirus, PD-1, PD-L1, human herpesvirus, innate immunity

## Abstract

Kaposi’s sarcoma-associated herpesvirus (KSHV) is associated with the human malignancy Kaposi’s sarcoma and the lymphoproliferative disorders primary effusion lymphoma and multicentric Castleman’s disease. KSHV establishes lytic infection of monocytes *in vivo*, which may represent an important cellular reservoir during KS disease progression. KS tumors consist of latently infected endothelial cells; however, lytic phase gene products are important for KS onset. Early KS lesion progression is driven by proinflammatory cytokines supplied by immune cell infiltrates including T cells and monocytes. KSHV-infected monocytes may supply the lytic viral products and the inflammatory milieu conducive to KS tumor progression. To establish successful infection, KSHV extensively modulates the host immune system. KSHV antigens activate both innate and adaptive immune responses including KSHV-specific T cells, but lifelong infection is still established. Programmed death ligand 1 (PD-L1) is a prosurvival cell surface protein that suppresses T-cell-mediated killing. PD-L1 is variably present on various tumor cells and is a targetable marker for cancer treatment. We show that KSHV infection of human monocytes increases PD-L1 expression and transcription in a dose-dependent manner. We also saw evidence of lytic gene expression in the KSHV-infected monocytes. Intact KSHV is needed for full PD-L1 response in human monocytes. KSHV induces a general proinflammatory cytokine milieu including interleukins 1α, 1β, and 6, which have been implicated in early KS lesion progression. KSHV-mediated PD-L1 increase may represent a novel mechanism of KSHV-mediated immune modulation to allow for virus survival and eventually malignant progression.

## IMPORTANCE

KSHV is the etiologic agent of Kaposi’s sarcoma and the lymphoproliferative disorders primary effusion lymphoma and multicentric Castleman’s disease. Programmed death ligand 1 (PD-L1) is an immunosuppressive cell surface marker that inhibits T cell activation. We report that KSHV infection of primary human monocytes upregulates PD-L1 transcription and protein expression. Analysis of the cytokine and chemokine milieu following KSHV infection of monocytes revealed that KSHV induces interleukins 1α, 1β, and 6, all of which have been implicated in KS development. Our work has identified another potential immune evasion strategy for KSHV and a potential target for immunotherapy of KSHV-derived disease.

## INTRODUCTION

Kaposi’s sarcoma-associated herpesvirus (KSHV) is associated with the endothelium-derived malignancy Kaposi’s sarcoma (KS) and the B cell lymphoproliferative disorders primary effusion lymphoma (PEL), multicentric Castleman’s disease (MCD), as well as cases of germinotropic lymphoproliferative disorder (GLD) (1–4). KS remains the most common AIDS-defining malignancy and the leading cause of cancer in sub-Saharan African men (5). KSHV-associated disease primarily manifests in immunocompromised patients such as solid organ transplant recipients, HIV-positive patients, and the elderly (5). A competent immune response is typically sufficient to prevent KSHV-mediated malignancies in the vast majority of lifelong KSHV infections. However, the immune system may also aid in early KS lesion progression, since a heavy immune cell infiltration and a proinflammatory cytokine milieu are present in early KS lesions and aid in lesion progression (6, 7). Although reconstitution of the host immune system via highly active antiretroviral therapy (HAART) in AIDS-KS or change of immunosuppressive regimens in iatrogenic KS often leads to clinical regression of KS (6), an improper immune response in immune reconstitution inflammatory syndrome (IRIS) during HAART-initiated repopulation of CD4^+^ T cells can lead to KS lesion progression (6). Therefore, a proper balance of immune response is vital for control of KSHV and prevention of its downstream malignancies. Programmed death ligand 1 (PD-L1) (also called B7-H1 and CD274) is an immunomodulatory cell surface protein, which interacts with the programmed death 1 (PD-1) (CD279) receptor on activated T, B, myeloid, and natural killer (NK) cells to induce a suppressive costimulatory signal (8–16). T cell receptor (TCR) signaling is blocked by PD-1–PD-L1 engagement, which leads to T cell anergy and immune tolerance. PD-L1 is constitutively expressed by a wide range of cells and is important in peripheral immune tolerance (9, 12, 17–20). PD-L1 is also induced by both type 1 and 2 interferons (19, 20). Chronic PD-1–PD-L1 interaction results in T cell exhaustion and is utilized by many viruses, including HIV, hepatitis C virus (HCV), and HBV, to induce an immune tolerant environment (21–23). A specific T cell response is generated to both KSHV latent and lytic antigenic proteins; however, it is apparent that KSHV-infected cells are not effectively cleared, since the virus establishes lifelong infection (24, 25). In addition to infections that induce increases in the PD-1–PD-L1 axis, cancer cells also upregulate PD-L1 levels (26–30). Importantly, therapeutic antibodies disrupting the PD-1–PD-L1 interaction have shown efficacy in treating PD-L1-overexpressing cancers (31–34).

KSHV is tropic for monocytes both *in vivo* and *in vitro* (35–40). Within KS, the primary tumor is composed of latently infected spindle cells combined with a small subset of lytic cells and inflammatory cell infiltrate. KS tumor progression is theorized to be dependent on proinflammatory cytokines. Cytokine involvement is suggested by the high dependency of KS spindle cells *in vitro* on cytokines for growth, exposure to these required cytokines *in vitro* induces a morphological shift to the classic spindle KS shape in endothelial cells, and high levels of proinflammatory cytokines including interleukin 1α (IL-1α), IL-1β, and IL-6 are found within the KS tumor microenvironment (7). The contribution of KSHV lytic replication to KS tumor development was demonstrated when AIDS patients were administered ganciclovir, a lytic phase inhibitor of herpesviruses, and KS incidence was significantly reduced (41). The lytic phase of KSHV infection results in the production of infectious virus and also induces the expression of all viral genes, which include viral homologues of cytokines and chemokines (5). Thus, lytic infection may be both inducing inflammation and reseeding tumor cells. Monocytes have been found to support KSHV lytic replication and may represent an important reservoir of virus during KS development (36). KS is the most common AIDS-defining cancer and coinfection of monocytes with KSHV and HIV leads to higher HIV replication (5, 37). Therefore, monocytes may play an important role in KSHV-associated disease. Previous studies have found that KSHV-positive PEL and GLD tumor cells are highly PD-L1 positive (42, 43) and that KS patient NK cells have increased PD-1 levels (44). Other infection events such as chronic infection of HIV and HBV also result in increased PD-L1 levels on CD14^+^ monocytes, which may establish an immune tolerant environment to allow for long-term infection (23, 45). A similar situation may be occurring with KSHV infection.

KSHV modulates the host immune system to successfully establish lifelong infection. During both the lytic and latent phases of KSHV’s life cycle, immunomodulatory genes are expressed. KSHV is able to disrupt a wide range of immune responses from innate immune sensing molecules, including Toll-like receptors, Nod-like receptors, and retinoic acid-inducible gene I product (RIG-I)-like receptors, to adaptive immune mechanisms, such as downregulation of major histocompatibility complex I (MHC-I) antigen presentation and expression of immunosuppressive viral cell surface homologues (46). Increased expression of PD-L1 may represent a novel pathway of immune suppression facilitated by KSHV.

We report that *de novo* infection of primary monocytes with KSHV induces increased PD-L1 levels through increased PD-L1 gene transcription. We also found evidence of lytic gene expression in the KSHV-infected monocytes. UV-inactivated KSHV failed to induce similar PD-L1 increases, suggesting that infection with KSHV is driving PD-L1 increase. Infection with increasing doses of KSHV correlate with increasing PD-L1 surface expression and transcription. KSHV-infected monocytes produce a general proinflammatory response similar to the KS tumor microenvironment cytokines with enrichment of IL-1α, IL-1β, and IL-6 compared to uninfected control cells. Overall, these data suggest that KSHV infection of monocytes may promote an immune tolerant environment and a cytokine milieu potentially allowing for both viral evasion and oncogenic progression.

## RESULTS

### KSHV infection upregulates PD-L1 surface expression on monocytes.

**FIG 1:**
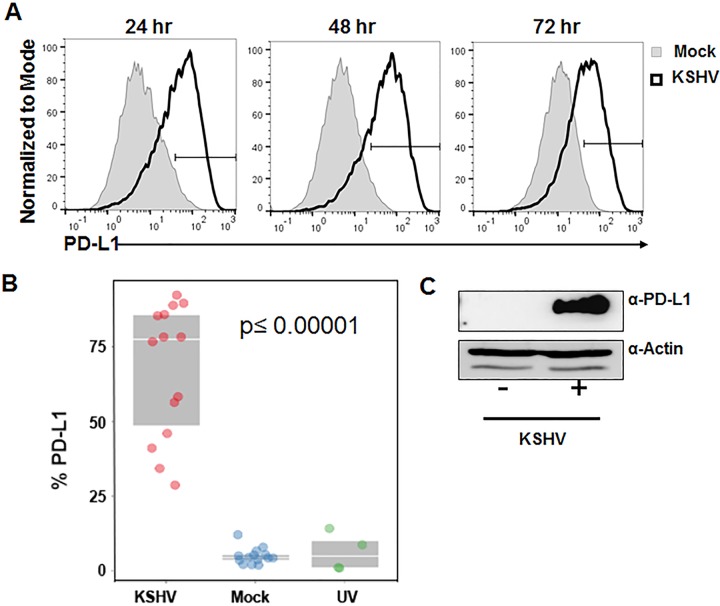
PD-L1 protein expression following KSHV infection. Primary human monocytes were isolated from buffy coats, and 5 × 10^6^ monocytes per well were infected with KSHV (2 × 10^9^ genomes/well). The cells were harvested at 24, 48, or 72 h postinfection and stained for CD14 and PD-L1, and expression of these markers was measured by flow cytometry. Cells were gated on forward scatter, side scatter, and CD14^+^. Histograms indicate the gate of PD-L1-positive cells with the gray-shaded histogram showing the percentages of mock-infected cells and the histogram outlined by the thick black line showing the percentages of KSHV-infected cells. (A) Representative histogram of the general trend from 14 independent donors. (B) Percent CD14^+^ cells expressing PD-L1 at 24 to 48 h after exposure to KSHV versus mock treatment or exposure to UV-treated KSHV (UV) for 48 h. The values between the KSHV-infected and mock-infected cells or the KSHV-infected and UV-inactivated KSHV-infected cells were significantly different (*P* ≤ 0.00001) by ANOVA *posthoc* comparison adjusted for multiple comparison by Tukey’s honestly significant difference test (HSD). (C) PD-L1 protein expression by immunoblotting at 36 h after KSHV infection of 3 × 10^6^ monocytes per well.

As KSHV has evolved multiple strategies for evasion of the immune response, we hypothesized that KSHV may modulate the PD-1–PD-L1 pathway. Monocytes are permissive for KSHV infection (35) and express PD-L1 (11). Therefore, we investigated whether KSHV infection of monocytes could alter expression of PD-L1. Primary human monocytes were isolated using negative selection from human peripheral blood mononuclear cells (PBMCs). The monocytes were then infected with intact KSHV, ultraviolet (UV) light-inactivated KSHV, or PBS (mock infected). Cells were harvested at 24, 48, or 72 h postinfection (hpi), and PD-L1 levels were measured via cell staining and flow cytometry. This analysis was performed with 14 different human donors at multiple time points after KSHV infection. CD14-positive monocytes show increased PD-L1 surface expression following KSHV infection at 24 hpi through 72 hpi (Fig. 1A). Intact KSHV is necessary for the increase in PD-L1 following infection (Fig. 1B). Whole-cell lysates harvested at 36 hpi show increased PD-L1 protein expression following KSHV infection compared to mock-infected cells (Fig. 1C). We consistently observed an increase in PD-L1 protein expression in CD14-positive cells compared to mock-infected cells from all donors (Table 1). The fold increase varies from 5.8-fold to more than 106-fold depending on the individual donor. These data suggest that infection of monocytes with KSHV results in a rapid and sustained increase in expression of the immunomodulatory protein PD-L1.

**TABLE 1:** Percentages of cells expressing PD-L1 following KSHV primary infection at 24, 48, and 72 h postinfection

Time point (hpi)	% cells expressing PD-L1 in:		Fold change
	Mock-infected cells	KSHV-infected cells	
24 h	5.26	58.3	11.08
	1.93	41.1	21.30
	4.33	34.3	7.92
	5.43	89.6	16.50
	3.58	85.8	23.97
	4.53	88.9	19.62
	3.77	92.3	24.48
	12.1	85.4	7.06
	2.06	76.7	37.23
	2.13	28.7	13.47
	7.94	46.0	5.79
	6.67	56.4	8.46
	4.31	78.3	18.17

48 h	4.61	74.6	16.18
	7.33	63.5	8.66
	0.23	24.4	106.09
	5.03	78.3	15.57
	3.78	62.4	16.51
	5.62	90.4	16.09

72 h	4.50	54.1	12.02
	6.60	88.2	13.36
	4.38	86.8	19.82
	2.37	44.1	18.61
	2.10	77.7	37.00
	4.68	32.0	6.84
	7.22	41.7	5.78
	4.47	82.4	18.43

**FIG 2:**
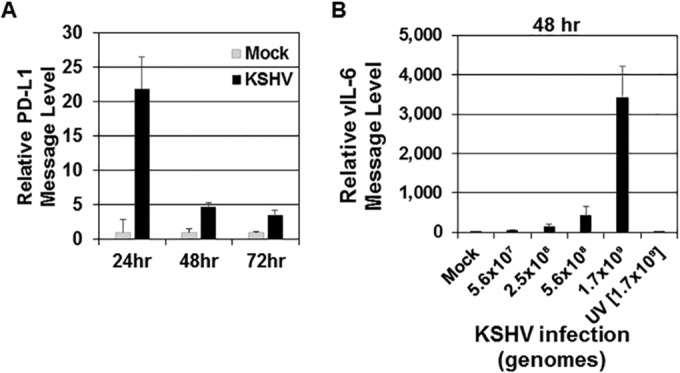
Levels of PD-L1 transcripts increase following KSHV infection. Human monocytes (5 × 10^6^ monocytes/well) were infected with KSHV (1 × 10^9^ genomes/well) (A and B) or UV-inactivated KSHV (B) for 24, 48, or 72 h. Total KSHV genomes were calculated prior to infection for panel B, and different amounts of KSHV genomes per well (5.6 × 10^7^, 2.5 × 10^8^, 5.6 × 10^8^, and 1.7 × 10^9^) were used. UV-inactivated KSHV genomes used were 1.7 × 10^9^ per well. At each time point, a mock-infected control was also harvested. After harvest, RNA was isolated from cells and reverse transcribed, and cDNA levels were measured through quantitative real-time PCR to assess either PD-L1 (A) or KSHV-encoded viral interleukin-6 (vIL-6) (B) message levels. RNA levels were normalized to β-actin and are represented as fold increase over mock-infected values at each time point. The data are representative plots of single experiments but represent the general trend of 11 donors for PD-L1 (24 hpi) transcript levels and 5 donors for vIL-6 (48 hpi) transcript levels. Error bars represent standard deviations of the fold change values for three technical replicates.

As PD-L1 expression on the surfaces of monocytes increased in KSHV-infected cells compared to mock-infected cells, we hypothesized that this may be due to an increase in PD-L1 transcription. RNA was harvested from mock-infected and KSHV-infected cells at 24, 48, and 72 h, reverse transcribed, and subjected to real-time PCR (RT-PCR) to assess PD-L1 transcript levels. All PD-L1 transcript levels were normalized to β-actin message. PD-L1 transcription peaks at 24 h postinfection and then tapers off at 48 and 72 h postinfection (Fig. 2A). The general trend was observed for 14 donors with various infectious doses and time points analyzed; 24 hpi (*n* = 14), 48 hpi (*n* = 9), and 72 hpi (*n* = 8). These data suggest that infection with KSHV increases PD-L1 transcription at early time points and that while the protein level (Fig. 1C) remains high, message levels decrease over time.

To ensure that the increase in PD-L1 message and level of expression is due to primary KSHV infection, the level of KSHV RNA present inside the infected monocytes was assessed. RNA was extracted from monocytes at 48 h after infection with either intact KSHV or UV-inactivated KSHV. The levels of viral interleukin-6 (vIL-6) were quantified via real-time PCR for message levels at 48 h postinfection (47). KSHV vIL-6 at 48 hpi showed increasing levels correlated with increasing dosages of intact KSHV (Fig. 2B) (24 and 72 hpi data not shown). KSHV transcripts were virtually undetected in mock-infected or UV-inactivated KSHV-infected cells.

### KSHV displays lytic infection at 48 h postinfection in monocytes.

**FIG 3:**
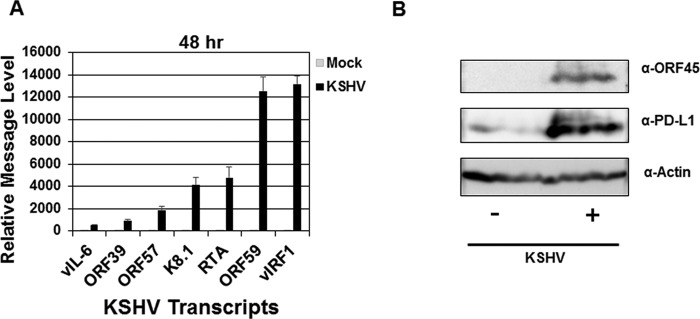
KSHV establishes lytic infection of human monocytes at 48 h postinfection. Human monocytes (5 × 10^6^ monocytes/well) were infected with KSHV (8.7 × 10^7^ genomes/well) for 48 h. At each time point, a mock-infected control was also harvested (A and B). (A) After harvest, RNA was isolated from cells and reverse transcribed, and cDNA levels were measured using quantitative real-time PCR to assess KSHV-encoded viral interleukin-6 (vIL-6), open reading frame 39 (ORF39), ORF57, ORF59, K8.1, viral interferon regulatory factor 1 (vIRF1), and replication and transcription activator (RTA) message levels as normalized to β-actin and represented as fold increase over mock-infected values. Error bars represent the standard deviations of fold change values for three technical replicates. (B) Immunoblot of KSHV viral protein ORF45 at 48 h postinfection. The data are representative plots of single experiments but represent the general trend of at least two (A) and four (B) independent donors and experiments.

Similar to other herpesviruses, KSHV infection displays either a latent or lytic life cycle (48). We infected primary human monocytes with KSHV, and cells were harvested at 48 hpi. We determined the levels of various lytic KSHV transcripts via RT-PCR including the following: vIL-6, open reading frame 39 (ORF39), ORF57, ORF59, K8.1, viral interferon regulatory factor 1 (vIRF1) and replication and transcription activator (RTA) (48–50) (Fig. 3A). Further, we determined the protein level of the lytic KSHV ORF45 protein via immunoblotting (51) (Fig. 3B). The presence of both lytic transcripts and protein are highly suggestive of active KSHV lytic infection.

### PD-L1 response is dependent on infection with intact KSHV.

**FIG 4:**
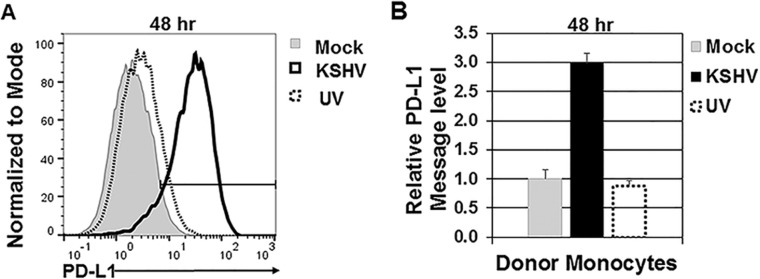
Infection with intact KSHV results in increased PD-L1 expression. Primary human monocytes (5 × 10^6^ monocytes/well) were infected with either intact or UV-inactivated KSHV (9.5 × 10^8^ genomes/well) and harvested at 48 h postinfection (A and B). (A) Cells were gated on forward scatter, side scatter, and CD14^+^. Histograms indicate the percentages of PD-L1-positive cells with the percentages for mock-infected cells, UV-inactivated KSHV-infected cells, and high-dose KSHV-infected cells. The results shown in panel A are representative of the results from four independent human donors. (B) RNA was isolated from cells and reverse transcribed, and cDNA levels were measured through quantitative real-time PCR to assess PD-L1 message levels. RNA levels were normalized to β-actin and represented as fold increase over mock-infected monocytes. Error bars represent standard deviations of fold change values for three technical replicates. The data are representative of the general trend of four independent donor experiments.

We investigated the ability of UV-inactivated KSHV virions to increase PD-L1 levels at 48 h postinfection (Fig. 4A). PD-L1 surface protein levels were increased by intact KSHV virions; however, UV-inactivated KSHV did not induce an increase in PD-L1 similar to the level induced by intact KSHV (Fig. 4A). Infection with UV-inactivated KSHV resulted in PD-L1 transcript levels similar to those of mock-infected cells (Fig. 4B).

### KSHV-mediated increase in PD-L1 expression is dose dependent.

**FIG 5:**
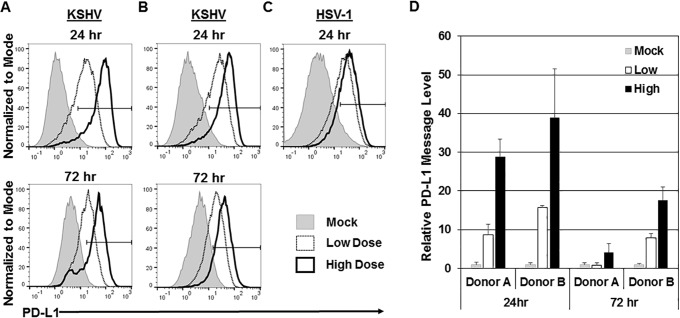
Increase in PD-L1 expression is dose dependent. Primary human monocytes (5 × 10^6^ monocytes/well) were infected with a low dose of KSHV (3 × 10^8^ genomes/well) or a high dose of KSHV (2.1 × 10^9^ genomes/well) or mock infected and harvested at 24 and 72 h postinfection. (A and B) Cells were stained for CD14 and PD-L1, and expression of these markers was measured by flow cytometry. Cells were gated on forward scatter, side scatter, and CD14^+^. Histograms indicate the gate of PD-L1-positive cells with percentages for mock-infected cells, low dose of KSHV-infected cells, and high dose of KSHV-infected cells shown. Panels A and B show representative data from donors from seven independent experiments. (C) Monocytes from a donor were infected with low-dose HSV-1 (MOI of 0.02) and high-dose HSV-1 (MOI of 0.2). (D) RNA was isolated from mock- and KSHV-infected cells and reverse transcribed, and cDNA levels were measured through quantitative real-time PCR to assess PD-L1 message levels. RNA levels were normalized to β-actin and represented as fold increase over the values for mock-infected monocytes. Error bars represent standard deviations of the fold changes for three technical replicates. The data are representative of the general trend of seven independent donor experiments.

We next investigated whether there was a correlation between the dose of virus used for infection and the levels of PD-L1 and cytokine production. Monocytes isolated from the same donor were infected with a low dose of KSHV (3 × 10^8^ genomes/well) and a high dose of KSHV (2.1 × 10^9^ genomes/well). At both 24 and 72 h postinfection, the cells that were infected with the higher dose of virus expressed higher levels of PD-L1 on the cell surface than the cells infected with the low dose of virus (Fig. 5A). This effect was observed with multiple donors (Fig. 5B). To better understand the general monocytic PD-L1 response to herpesviruses, we also infected monocytes with herpes simplex virus 1 (HSV-1) at multiplicities of infection (MOIs) of 0.02 and 0.2. The PD-L1 response to HSV-1 showed a similar dose-dependent increase to increasing HSV-1 doses (Fig. 5C). Moreover, an increase in PD-L1 protein was accompanied by an increase in PD-L1 message in infected cells compared to mock-infected cells (Fig. 5D). Again, the message increase was variable between donors, but the general trend remained the same over 7 donors. These data suggest that increased doses of KSHV and HSV-1 correlate with the increase in PD-L1 transcription and protein production.

### Multiple cytokines and chemokines are induced in monocytes following KSHV infection.

**FIG 6:**
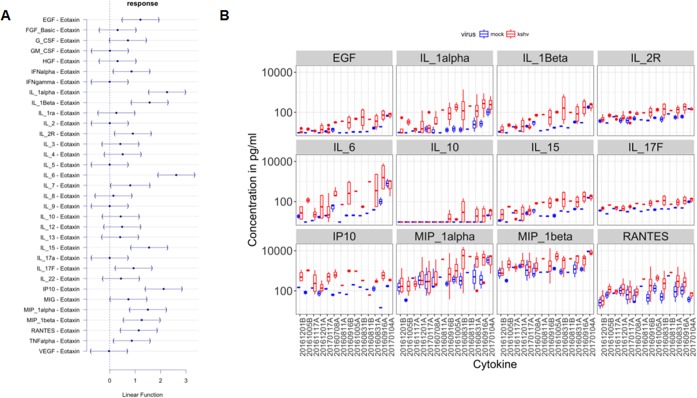
Cytokine response following KSHV infection. Supernatants from KSHV-infected and mock-infected monocytes were harvested at 24, 48, and 72 h postinfection. Supernatants were precleared and then subjected to the human cytokine magnetic 35-plex panel. The average raw values (in picograms per milliliter) of 35 analytes were measured. (A) Multivariate analysis after individual enrichment compared to matched mock value for the same donor was calculated, and data were Z standardized. The relative response on the horizontal axis compared to eotaxins is shown, which did not change in any of the experiments after adjustment for multiple comparisons by Dunnett’s *posthoc* test. The 95% confidence intervals (95% CI) of the response variables are also shown by the bars. 95% CIs that do not include zero are considered significant at *P* ≤ 0.05. (B) A box-and-whisker plot of cytokine levels in picograms per milliliter on the vertical axis in the supernatant of KSHV-infected and mock-infected monocytes for a group of cytokines that significantly and consistently increased upon KSHV infection. Note that the vertical axis is on a log_10_ scale. The horizontal axis indicates the different donors. The donors are ordered by mean enrichment to KSHV infection and not by unique identifier. The median values (horizontal bars), first and third quartile boundaries (boxes), and the 1.5× interquartile ranges (vertical bars) are shown for the cytokines measured shown at the top of each subpanel. Blue indicates mock-infected samples, and red indicates KSHV-infected samples. Note that some donors were exposed to different concentrations of virus, and measurements were taken at different time points (24, 48, and 72 h) after infections. These are aggregated here and explain some of the variation. The other 23 analytes in the human cytokine magnetic 35-plex panel did not show consistent changes upon KSHV infection. MCP-1 was excluded from the analysis, as the readings were consistently above the upper limit of the assay. A total of 14 independent donors were used.

PD-L1 expression can be driven by cytokine signaling pathways (52). Further, cytokines and chemokines play a role in KS tumor progression, particularly early in lesion development (6, 7). Therefore, we determined the production of a panel of cytokines and chemokines by KSHV-infected monocytes at 24, 48, and 72 h postinfection. The average cytokine levels in 14 donors at 24 h postinfection are shown in Table 2. At first glance, the levels of nearly all of the 35 analytes tested were increased in response to KSHV infection in monocytes compared to mock-infected monocytes (Table 2). In time course experiments, cytokines that were increased at 24 h postinfection exhibited higher levels at 48 and 72 h (data not shown). Cytokines that were increased at lower levels of KSHV infectious dose exhibited higher levels at higher levels of infectious dose (data not shown). Yet, there was also considerable random variation among donors, as would be expected for experiments with primary cells. To account for the random variation and experimental variation, we used multivariate analysis of variance (ANOVA). To account for multiple comparisons, we used Dunnett’s posthoc test on fold enrichment scores calculated on matched infected and mock-infected cells from the same donor. Furthermore, the data were Z standardized to allow comparison across different ranges of responses. Figure 6A shows the relative response on the horizontal axis compared to eotaxin, which did not change in any of the experiments; epidermal growth factor (EGF), alpha interferon (IFN-α), IL-1α, IL-1β, IL-6, IL-15, IL-17F, and IL-2 receptor (IL-2R) and the chemokines IP-10 (interferon-inducible protein of 10 kDa), MIP-1α (macrophage inflammatory protein 1α), MIP-1β, RANTES (*r*egulated on *a*ctivation, *n*ormal *T* cell *e*xpressed and *s*ecreted), and alpha tumor necrosis factor (TNF-α) changed significantly. Raw concentrations for significantly changed cytokines are shown in Fig. 6B for each of the donors. The changes in IL2R and IL-17F were minimal but consistent across donors and conditions. IL-10 increased in about half of all donors. Whether IL-6, MIP-1α, MIP-β, or RANTES Luminex beads exhibit cross-reactivity to the respective viral homologues is not known.

**TABLE 2:** Average increase in cytokines and chemokines 24 h after KSHV infection

Cytokine or chemokine^a^	Avg level (pg/ml) of cytokine or chemokine in:		Fold change
	Mock-infected cells	KSHV-infected cells	
FGF-Basic	14.60	21.01	1.44
IL-1β	29.02	195.31	6.73
G-CSF	70.86	166.38	2.35
IL-10	4.11	29.95	7.29
IL-13	9.68	19.18	1.98
IL-6	61.36	1,479.86	24.12
IL-12	16.56	30.87	1.86
RANTES	185.34	636.82	3.44
Eotaxin	0.58	0.79	1.35
IL-17A	2.55	5.22	2.04
MIP-1α	1,227.75	5,712.83	4.65
GM-CSF	0.56	3.10	5.57
MIP-1β	2,356.85	8,056.80	3.42
IL-15	35.06	150.26	4.29
EGF	14.18	60.95	4.30
IL-5	0.85	2.46	2.91
HGF	84.61	154.75	1.83
VEGF	4.18	5.82	1.39
IL-1β	31.48	392.39	12.46
IFN-γ	2.37	4.02	1.69
IL-17F	43.17	112.89	2.62
IFN-α	37.28	73.32	1.97
IL-9	1.36	3.56	2.62
IL-1ra	2,676.17	3,683.13	1.38
TNF-α	25.26	149.10	5.90
IL-3	6.22	17.14	2.76
IL-2	1.28	4.29	3.36
IL-7	10.54	31.28	2.97
IP-10	112.69	603.13	5.35
IL-2R	66.42	159.30	2.40
IL-22	32.88	53.35	1.62
MIG	9.51	24.41	2.57
IL-4	9.47	20.65	2.18
IL-8	27,310.93	29,531.38	1.08

a FGF, fibroblast growth factor; G-CSF, granulocyte colony-stimulating factor; GM-CSF, granulocyte-macrophage colony-stimulating factor; EGF, epidermal growth factor; HGF, hepatocyte growth factor; VEGF, vascular endothelial growth factor; MIG, monokine induced by gamma interferon.

Interestingly, the levels of nearly all of the 35 analytes tested were increased in response to KSHV infection in monocytes compared to mock-infected cells (Table 2). Monocyte chemotactic protein 1 (MCP-1) was included in the assay plate; however, regardless of conditions, readings regularly exceeded upper threshold and were excluded from analysis. Finally, KSHV increases IP-10 at each time point (Table 2), which correlates with our previous report that KSHV can activate Toll-like receptor 3 (TLR3), resulting in the induction of IP-10 downstream of TLR3 signaling (39, 53).

## DISCUSSION

In this report, we show that KSHV infection of human primary monocytes results in an increase in PD-L1 expression. This increase was at the transcriptional level, and although transcript levels decreased over time, protein levels remain increased at least until 72 h after primary infection with KSHV. We show that at 48 h postinfection of monocytes, KSHV expresses multiple lytic transcripts and the lytic protein ORF45, suggesting establishment of an overall lytic phase of infection. Cells that were exposed to increasing amounts of virus exhibited increased KSHV vIL-6 transcription as well as increased PD-L1 transcription and protein expression. In addition to an increase in PD-L1 expression, infected cells also increased production of a variety of generally proinflammatory cytokines and chemokines. Together these data suggest that KSHV induces an immune response in cells and one of the potential mechanisms that KSHV may utilize to decrease immune responses is through upregulation of the immunomodulatory protein PD-L1.

KSHV-mediated expression of PD-L1 may be an additional mechanism among many already identified for KSHV evasion of host immune responses. It is critical that KSHV avoid detection in order to maintain lifelong infection within the host. To this end, mechanisms for blockade of many major immunological pathways have already been identified including the host complement system, type I interferons, Toll-like receptors, Nod-like receptors, and cytosolic DNA sensors (54). For many of these pathways, KSHV encodes homologues of cellular proteins which inhibit the pathway such as vIL-6, CD200, and vIRFs. KSHV also expresses some mechanisms to evade adaptive immune responses, such as downregulation of MHC-I (54).

The role of PD-1–PD-L1 in acute infection is not as widely studied as in the chronic infection setting (55). Our data examine PD-L1 modulation during acute infection. Due to the fact KSHV establishes lifelong infection in the host, this means that primary infection of naive cells takes place sporadically throughout the life of the host following bouts of viral reactivation from latency (5). Spontaneous production of viral progeny likely results in periodic infection of naive cells such as monocytes. Although our data show that PD-L1 levels increase in acute KSHV infection, it is possible that repeated primary infection may result in chronic PD-L1 upregulation in monocytes. Our data also reveal a potential mechanism of host evasion utilized by KSHV to establish infection. A previous report has shown that KS patient NK cells have increased PD-1 surface expression and show an exhausted phenotype upon stimulation *in vitro* (44). Further, inflammatory infiltrates within the KS tumor microenvironment were shown to express PD-L1 (44). As monocytes are a common infiltrate of KS tumors, our data suggest that KSHV primary infection of monocytes may be in part contributing to increases in PD-L1 expression and downstream PD-1–PD-L1-induced NK cell exhaustion. Further, KS tumor progression is highly dependent on inflammatory cytokines in the tumor microenvironment. We report that KSHV induces an overall proinflammatory cytokine milieu including IL-1α, IL-1β, and IL-6, which have all been reported to be important for KSHV-derived malignancies (6, 7). Finally, previous reports have also shown that PEL and GLD tumor cells can display increased PD-L1 expression (42, 43).

As PD-L1 and PD1 are overexpressed in a subset of cancers and therapies to block these pathways have helped to reduce tumor burdens, expression of PD-L1 in KSHV-infected cells could be of great interest to reduce KSHV persistence and associated malignancies. Further investigation of KSHV-modulated PD-L1 expression may reveal individual viral genes responsible for upregulating PD-L1 and potential mechanisms for targeted treatment of KSHV-associated cancers.

## MATERIALS AND METHODS

### Monocyte isolation, virus generation, and infection.

Human monocytes were isolated from buffy coats (Gulf Coast Regional Blood Center) using the monocyte isolation kit II (Miltenyi Biotec) according to the manufacturer’s instructions. The CD14-positive monocytes were assayed for purity, and in all experiments, only a ≥85% pure monocyte population was used. The resulting monocytes were plated into six-well dishes at 5 × 10^6^ cells/well in RPMI medium.

Kaposi’s sarcoma-associated herpesvirus (KSHV) was isolated from iSLK.219 cells harboring latent recombinant KSHV.219 (rKSHV.219) (56). iSLK.219 cells were maintained in Dulbecco modified Eagle medium (DMEM) supplemented with 10% fetal bovine serum (FBS) (Cellgro), 1% penicillin and streptomycin (Pen-Strep), G418 (250 μg/ml), hygromycin (400 μg/ml), and puromycin (10 μg/ml) (56). To produce infectious virions, the medium was changed to DMEM containing 1% Pen-Strep, 10% FBS, and 3 μg/ml doxycycline, and 1 mM sodium butyrate (56). After 72 h, supernatant was harvested, and cell debris was pelleted and filtered through a sterile 0.45-μm filter. Virus was concentrated as previously described (57). UV inactivation of KSHV was performed as previously described (39).

Human monocytes were centrifuged with a range of KSHV doses from 4.2 × 10^7^ to 7.8 × 10^9^ genomes/well and 10 μg/ml Polybrene in serum-free RPMI medium at 2,500 rpm for 90 min at 30°C as previously described (39). Mock-infected cells were treated with an equivalent volume of phosphate-buffered saline (PBS), the buffer utilized to concentrate KSHV. Immediately following centrifugation, fetal bovine serum was added to the medium at a final concentration of 20%. The cells and medium supernatants were harvested at the times indicated.

### Flow cytometry.

Cells were stained with CD14 (61D3; eBioscience) and PD-L1 (29E.2A3; BioLegend) prior to analysis on a MACSQuant VYB cytometer (Miltenyi Biotec) and FlowJo software (Tree Star). Programmed death ligand 1 (PD-L1) levels were ascertained after gating on CD14.

### Immunoblots.

Cells were harvested, washed once with PBS, and then lysed. Lysis was mediated through two separate methods depending on cell number harvested. For samples less than or equal to 5 × 10^6^ monocytes, cells were resuspended in 40 microliters of a solution of 50 ml NP-40 buffer (0.1% NP-40, 150 mM NaCl, 50 mM Tris-HCl [pH 8.0] containing a proteinase inhibitor tablet [Roche], 30 mM β-glycerophosphate, 50 mM NaF, and 1 mM Na_3_VO_4_) and subsequently frozen and thawed once to complete lysis. For samples more than 5 × 10^6^ monocytes, cells were resuspended in 2× Laemmli sample buffer and simultaneously boiled and vortexed at 1,200 rpm in a ThermoMixer (Eppendorf) until the pellet was no longer visible (10 to 20 min). Equal amounts of protein were loaded per lane, resolved by SDS-PAGE, and then transferred to a nitrocellulose membrane. The antibodies used were PD-L1 (E1L3N; Cell Signaling Technology), ORF45 (2D4A5; Thermo Fisher), and actin (C-11; Santa Cruz).

### Nucleic acid isolation and real-time PCR.

RNA was isolated using RNeasy Micro kit (Qiagen) and reverse transcribed with SuperScript III reverse transcriptase (Invitrogen) and oligo(dT) (Invitrogen). Real-time PCR was performed on a QuantStudio 6 Flex (Applied Biosystems) with PowerUp SYBR green PCR master mix (Applied Biosystems). The PCR was carried out with 1 cycle of 50°C for 2 min and 95°C for 10 min, followed by 40 cycles of 95°C for 15 s and 60°C for 1 min. All fold activations were normalized to β-actin. The primer sequences used for β-actin and PD-L1 were as follows: for β-actin, forward, 5′-TCATGAAGTGTGACGTGGACATC, and reverse, 5′-CAGGAGGAGCAATGATCTTGATCT (58); for PD-L1, forward, 5′-GGTGCCGACTACAAGCGAAT, and reverse, 5′-AGCCCTCAGCCTGACATGTC (59). Proprietary PD-L1 primers (SA Biosciences) were also used and showed threshold cycle (*C*_*T*_) values similar to those of the aforementioned primers. The forward and reverse primers for cytokines, chemokines, and other targets follow: for viral IL-6 (vIL-6), forward, 5′-CGGTTCACTGCTGGTATCT, and reverse, 5′-CAGTATCGTTGATGGCTGGT (47); for open reading frame 39 (ORF39), forward, 5′-GGTTTCCCCTGCTACTTCAA, and reverse, 5′-CATGCTTGGCCCGATATAC; for ORF57, forward, 5′-TGGACATTATGAAGGGCATCCTA, and reverse, 5′-CGGGTTCGGACAATTGCT; for ORF59, forward, 5′-TTGGCACTCCAACGAAATATTAGAA, and reverse, 5′-CGGAACCTTTTGCGAAGA; for K8.1, forward, 5′-AAAGCGTCCAGGCCACCACAGA, and reverse, 5′-GGCAGAAAATGGCACACGGTTAC; for replication and transcription activator (RTA), forward, 5′-TGTAATGTCAGCGTCCACTC, and reverse, 5′-ATTGCTGGGCCACTATAACC; for viral interferon regulatory factor 1 (vIRF1), forward, 5′-CGTGTCCTTTGGTGAAACTG, and reverse, 5′-TCGGCATTATTTCGAGTACG (49).

### Cytokine and chemokine determination.

Supernatants were precleared and tested with a human cytokine magnetic 35-plex panel (Life Technologies) per the manufacturer’s instructions and analyzed with a MAGPIX instrument (Luminex).

### Statistical analysis.

All calculations were conducted using the R software package version 3.3.3 “Another Canoe” (released 6 March 2017) with the specific statistical tests indicated. Code is available upon request.
